# Toward Timely and Equitable Advanced Biomarker Testing for Patients with Metastatic Cancer in Canada

**DOI:** 10.3390/curroncol32030141

**Published:** 2025-02-27

**Authors:** Brandon S. Sheffield, Shantanu Banerji, Allen Chankowsky, Shaan Dudani, Sharlene Gill, Zuzanna Gorski, Shaqil Kassam, Cassandra Macaulay, Mita Manna, Kirstin Perdrizet, Ravi Ramjeesingh, Monika Slovinec D’Angelo, Filomena Servidio-Italiano

**Affiliations:** 1 William Osler Health System, Brampton, ON L6R 3J7, Canada; brandon.sheffield@williamoslerhs.ca (B.S.S.); shaan.dudani@williamoslerhs.ca (S.D.); kirstin.perdrizet@williamoslerhs.ca (K.P.); 2 Rady Faculty of Health Sciences, Department of Medical Oncology and Hematology, University of Manitoba, CancerCare Manitoba, Winnipeg, MB R3E 0V9, Canada; sbanerji@cancercare.mb.ca; 3 Patient Advocate, Member of the ASCO TAPUR molecular tumour board, Toronto, ON, Canada; allen@allenchankowsky.com; 4 BC Cancer Agency, Vancouver, BC V5Z 4E6, Canada; sgill@bccancer.bc.ca; 5 Anatomical Pathology and Laboratory Medicine, QEII Health Sciences Centre, Halifax, NS B3H 1V8, Canada; zgors042@uottawa.ca; 6 Stronach Regional Cancer Centre, Newmarket, ON L3Y 2P9, Canada; skassam@southlakeregional.org; 7 Colorectal Cancer Resource & Action Network (CCRAN), Toronto, ON M4W 3E2, Canada; cassandra.m@ccran.org (C.M.); monslovinec@gmail.com (M.S.D.); 8 Saskatoon Cancer Centre, Saskatoon, SK S7N 4H4, Canada; mita.manna@saskcancer.ca; 9 Division of Medical Oncology, QEII Health Sciences Centre, Halifax, NS B3H 2Y9, Canada; ravi.ramjeesingh@nshealth.ca

**Keywords:** molecular testing, biomarker testing, mutational testing, genomic testing, next generation sequencing, comprehensive genomic profiling, liquid biopsy, circulating tumor DNA (ctDNA), genomic medicine, precision medicine

## Abstract

The explosion in biomarker testing over the past two decades continues to transform cancer care in Canada and around the world. Precision medicine is supported by identifying actionable mutations that direct therapeutic choices, thus improving survival and quality of life, especially for patients with advanced/metastatic disease. In addition, our growing understanding of the genetic basis of cancer is advanced by research employing ever-expanding databases of genetic mutations, therapies and outcomes. Despite this promising progress, however, access to biomarker testing remains inequitable across Canada, to the detriment of patients. Several underlying factors contribute to this situation, including the need for investment in and standardization of laboratory medicine infrastructure and processes, and the lack of suitable methods for cost/benefit evaluations to inform funding decisions. In 2024, a Canadian conference brought together patients, clinicians, researchers, policy-makers and scientists to address “Equitable Access to Advanced Biomarker Testing for Canadian Metastatic Cancer Patients”. Two major themes arose from the conference: the urgent need to adopt comprehensive genomic profiling (CGP) as a standard of care across Canada, and the emerging role of liquid biopsy in accelerating access to biomarker testing for patients with advanced/metastatic cancer.

## 1. Introduction

### 1.1. Evolution of Biomarker Testing in Cancer Therapy in Canada

The past two decades have seen an explosion in the identification of biological markers for tumour mutations, and biomarker testing is rapidly becoming a standard of care for patients diagnosed with cancer in Canada. 

Biomarkers can indicate the normality of cell function, the aggressiveness of tumour growth, and predicted tumour response to drug therapies. They therefore play a valuable role by aiding in early detection, diagnosis, prognosis, treatment response, and personalized treatment approaches for cancer [1]. Biomarker testing particularly benefits patients with aggressive or advanced cancers, for whom therapeutic options are often limited. Biomarker testing can help to identify targeted systemic therapies [2,3]. Also, clinical trials are often the mainstay of treatment for late-stage cancer patients and inclusion criteria increasingly are based on biomarker profiles. 

Terminology in this paper follows consensus recommendations [4]. The term “biomarker testing” refers to tests that identify characteristics, targetable findings, or other test results originating from malignant tissue or blood. Testing can include single tests, panel tests, and multiplex panel tests (such as comprehensive genomic profiling, CGP, and next generation sequencing, NGS). 

#### The Value of Biomarker Testing

The scope of potential benefit to patients and to Canadians at large from biomarker testing is massive. An estimated 247,100 Canadians are expected to be diagnosed with cancer in 2024 [5]. Between 2018 and 2020 more than 1.3 million potential years of life were calculated to have been lost from cancer in Canada [6]. The economic burden of cancer in Canada was estimated to be $35 billion in 2024 [7].

From a system perspective, biomarker testing has the potential to improve the quality of cancer care while reducing costs. Total net public expenditures on cancer care are mainly driven by hospital-based services and costs are highest in the late stages of cancer [8] where biomarker testing plays an outsized role. By comparison, laboratory testing accounts for a tiny proportion of these costs. In Ontario, for example, lab testing represents just 0.25% of the total cost of cancer care to the province [8]. 

By advancing precision medicine, biomarker testing continues to enable accelerated drug development, bringing forward more therapeutic options and defining which patients are most likely to benefit from them. For complex diseases such as cancer, targeted therapies have become mainstays of treatment and biomarker testing has the potential to streamline approval processes for new drugs and their companion tests [9]. 

As the field of biomarker testing continues to evolve in Canada, calls to action have been voiced by cancer care specialists and patients for access to comprehensive genomic testing (CGP), faster approvals for a growing arsenal of targeted therapeutics, and for an integrated approach to their utilization.

### 1.2. Conference Organization

The Colorectal Cancer Resource & Action Network (CCRAN) hosted a conference to highlight the value of biomarker testing in optimizing cancer care and to elaborate on systemic barriers that impede patient access. Held on 20–21 June 2024, this was the second conference on biomarkers hosted by CCRAN, a not-for-profit patient organization that supports, educates and advocates on behalf of cancer patients and their families in Canada. The 2023 Biomarkers Conference report was previously published in this journal [10].

Participants in the 2024 Biomarkers Conference included 52 expert speakers from Canada, the United States, Europe and Australia; patient advocacy group leaders representing multiple tumour sites; and 553 registrants from 22 countries. 

The development of the meeting agenda, which consisted of 14 virtual educational and interactive sessions, was guided by an Expert Steering Committee (detailed in Appendix A).

The conference goals focused on 3 key areas:

- Challenges and barriers to timely and equitable access to biomarker testing in Canada;
- The potential of comprehensive genomic profiling (CGP); and
- The future of liquid biopsy in biomarker testing.

## 2. Challenges and Barriers to Timely and Equitable Access to Biomarker Testing

### 2.1. Identifying the Challenges and Barriers Experienced by Patients and Clinicians

Themes of the 2024 Biomarkers Conference built on the issues identified at the 2023 Biomarkers Conference. These were:

- Limited patient and clinician awareness of molecular/precision medicine options with need for formal education strategies;
- Failure of clinicians to discuss biomarker testing with patients;
- Lack of standardized testing and delays in reporting of results; and
- Intra- and inter-provincial disparities in access to biomarker testing, including impact of social determinants of health,

The publication resulting from the 2023 Biomarkers Conference was utilized as an advocacy tool by CCRAN in its meetings with provincial Ministries of Health to bring to their attention the barriers and challenges identified by conference participants.

The 2024 Biomarkers Conference built on these outputs by focusing discussions on gaining a deeper understanding of the issues for patients with advanced cancer. 

A consistent theme throughout the conference was the similarities in patient experiences across different cancer sites. This was exemplified by an online survey conducted in the spring of 2024 by CCRAN in collaboration with patient advocacy groups representing 24 tumour sites. The survey sought to understand the lived experiences of patients with metastatic or advanced cancer with respect to biomarker testing and CGP [11]. As shown in Table 1, survey findings from 159 cancer patients from across Canada reinforced the conclusions of the 2023 Biomarkers Conference.

This conference report is focused on two underlying factors contributing to the lack of timely and equitable access to biomarker testing in Canada, namely: fragmentation of care and disparities in public funding among provinces and institutions. The situation was summarized in a recent publication as follows: “The efficient and considered adoption of novel genomic medicine testing is hampered in Canada by the fragmented nature of health care oversight as well as by lack of clear and transparent processes to support rapid evaluation, assessment, and implementation of genomic tests” [9].

### 2.2. Fragmentation of Care

The absence of standardized and integrated protocols significantly hampers access to high quality biomarker testing in Canada. A personal experience cited during the conference illustrates the need for greater integration of care at the institutional level:

“*My husband was diagnosed with Stage IV rectal cancer at age 37 and was started on standard chemotherapy. A biopsy specimen was sent for biomarker testing in order to determine his eligibility for targeted therapies. I asked several times for the results and was eventually told that, because the sample was too small, a repeat biopsy was needed. Then, due to an oversight by the lab, analysis of the second sample was further delayed. In total, we waited 6 months after diagnosis for the results of the test, which delayed the decision about which [targeted drug therapy] to use.*”

This situation, which caused much stress and anxiety in circumstances that were totally unfamiliar to the patient and caregiver, was due to a lack of coordination within the healthcare system and between the patient and care team. The experience was echoed in a recent study of biomarker testing for colorectal cancer (CRC) among Canadian hospitals which found large discrepancies in timing of results, differences in prescribed treatments based on biomarker reports, and widely divergent quality of reporting [12]. 

At a broader level, conference participants asserted that a lack of harmonization of biomarker testing processes within and across jurisdictions creates barriers that contribute to inequities. Divergent processes and quality standards relate to, for example, reporting and data-sharing strategies; lists of tested biomarkers and assays; methodologies for obtaining the appropriate diagnostic specimens for tissue and liquid biopsy; and recruitment and training programs. 

### 2.3. Disparities in Funding

At the health system level, conference participants noted that disparities between provinces and institutions create inequities to access to biomarker testing. For example, for CRC and gynecologic cancers biomarker testing is publicly funded in Ontario only for genes for which there is a matched therapy. In Nova Scotia, testing using a 50-gene panel is now publicly funded for metastatic CRC and lung cancer but only upon request for hepatic cancer. Western provinces don’t pay for CGP. Quebec publicly funds all molecular tests ordered by physicians. In some provinces, expanded panels are available only in certain centres where funding is available from foundations or from private sources, however patients are usually unaware of these options. Access to CGP may be available through a clinical trial if a patient is eligible for inclusion and provided their treatment centre is participating in the study. The availability of local infrastructure and the influence of patient champions were also cited as factors contributing to access disparities.

Conference participants agreed that conventional health technology assessment (HTA) methods are not well equipped for evaluating biomarker tests and that this contributes to funding disparities. Innovative approaches are needed to address the uncertainties inherent in this evolving field. New models of evaluation are needed that are adaptable to new realities, as tests become cheaper and predictive analytics play an expanded role in directing testing and treatment.

## 3. The Value and Potential of Comprehensive Genomic Profiling

Comprehensive genomic profiling (CGP) uses next-generation sequencing (NGS) to analyze a panel of several hundred genes in a tumour tissue sample, consolidating biomarker detection into a single multiplex assay and eliminating the need for sequential testing. Comprehensive testing may provide actionable information to help guide clinical decisions and has the potential to reveal obscure targets which may have future utility.

### 3.1. Current Practices in Canada

CGP has been available for decades. Conference presenters noted that in Canada it is used only in specific situations where much is already known about mutations (such as in lung cancer where 50-gene “hotspot” panels are typically used) or in the metastatic cancer setting where standard therapies have been exhausted and the patient’s mutational profile may be used to assess their candidacy for clinical trials. 

In comparison, most major cancer treatment centres in the United States offer CGP to all Stage IV patients, however access is largely determined by the patient’s insurance status. Some patients in Canada access CGP through international private-payer laboratories, amplifying socioeconomic barriers to health care access.

### 3.2. Benefits of CGP

CGP serves the personalization of treatment choices because every patient’s tumour has a unique molecular makeup and individual tumours develop differently, and as such may be exploited using precision medicines. Furthermore, databases of testing results benefit research by expanding knowledge about various types of cancer and can inform drug development and HTA evaluation. 

CGP has had profound impacts for some patients. During the conference, Dr. David M. Thomas cited updated data from Australia based on the Cancer 2015 and MoST studies [13,14] which showed that treatments are now found for 70% of patients who have been tested [Thavaneswaran et al., unpublished], compared to only 5–10% ten years ago [Thomas et al., unpublished]. New data show that 37% of patients categorized as incurable actually have targets that may double survival if appropriate therapies are utilized [Thomas, personal communication].

A patient experience presented at the conference illustrates the potential and challenges of CGP in Canada. Diagnosed with terminal head and neck cancer in 2016, the patient asked for CGP testing but was told that it was unavailable. Navigating the healthcare system on his own, the patient travelled to the United States for testing and the results guided his treatment. Thankfully, for the past 8 years the patient has been in a prolonged remission and the cancer is now being managed as a chronic disease.

Although CGP is widely viewed as expensive, its costs are 10-fold less than those of treatments for advanced cancers [15,16]. 

**Figure 1:**
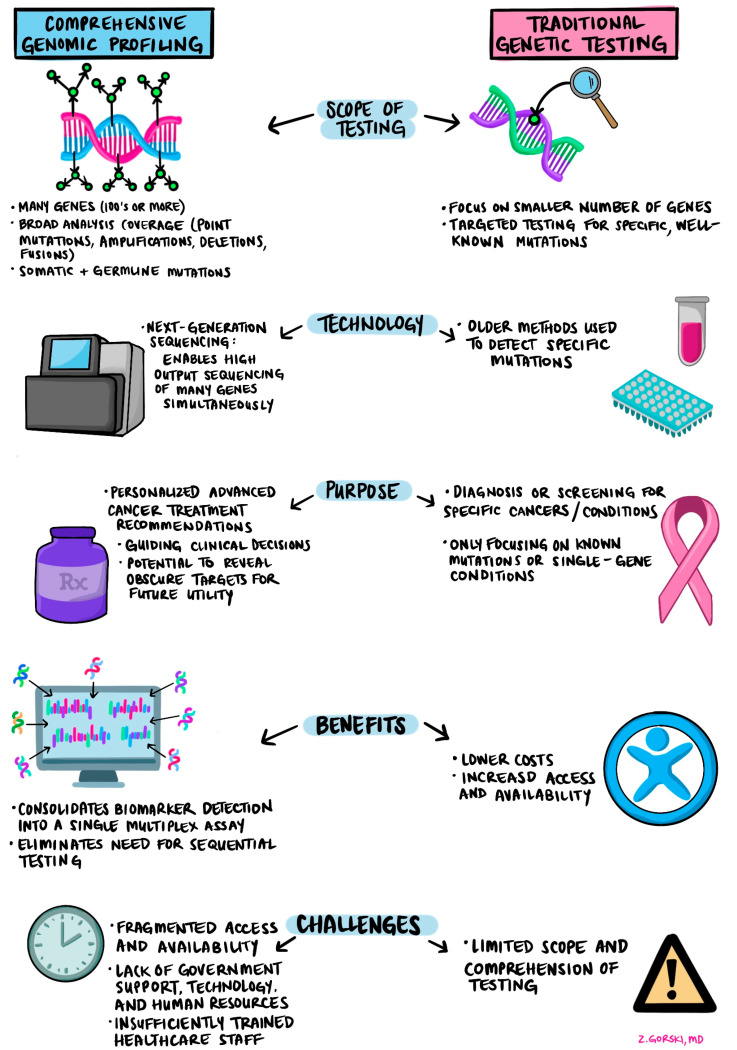
Comparison of Comprehensive Genomic Profiling (CGP) and traditional genetic testing.

Figure 1 summarizes the advantages and drawbacks of CGP and illustrates the relationship between CGP and traditional genetic testing.

## 4. The Value of Liquid Biopsy

Liquid biopsy analyzes for circulating biomarkers shed by tumour cells, such as cell-free nucleic acids, circulating tumour cells, proteins, and nucleosomes [17]. Information is provided on specific mutational signatures, tumour mutational burden, sequences, and epigenetic changes. Because it tests a mix of molecules released from various subclones in the tumour, liquid biopsy differs from tissue biomarker testing in that it captures the heterogeneity of a particular cancer at a point in time. The technique has multiple applications in cancer screening, biomarker profiling, detection of residual disease after curative-intent surgery, monitoring of treatment response, and monitoring of the clonal evolution of tumours.

### 4.1. Current Liquid Biopsy Practices in Canada

In Canada, adoption of liquid biopsy as a routine test has lagged behind the rapid uptake seen in the United States. Rather, liquid biopsy in Canada serves as an adjunct to molecular profiling of solid tissue samples. It is especially valuable as a rapid test where the timeliness of treatment is critical, such as in non-small cell lung cancer (NSCLC) where the mortality of untreated advanced disease is 4% per week [18,19]. It also benefits patients with other tumour types who are too ill or compromised to undergo tissue biopsy. Because access to CGP for NSCLC remains unequal across regions and is also sometimes limited by insufficient tissue samples and long turn-around times, a rapid plasma-based test can provide vitally important information quickly. 

The William Osler Health System in Ontario is one of the first cancer care centres to in-source liquid biopsy biomarker testing. A study of the concept of community hospital-based testing showed a median 3-day turnaround time to results, compared to approximately 14 days using traditional centralized reference labs [17]. 

### 4.2. Benefits and Costs of Liquid Biopsy

**Figure 2:**
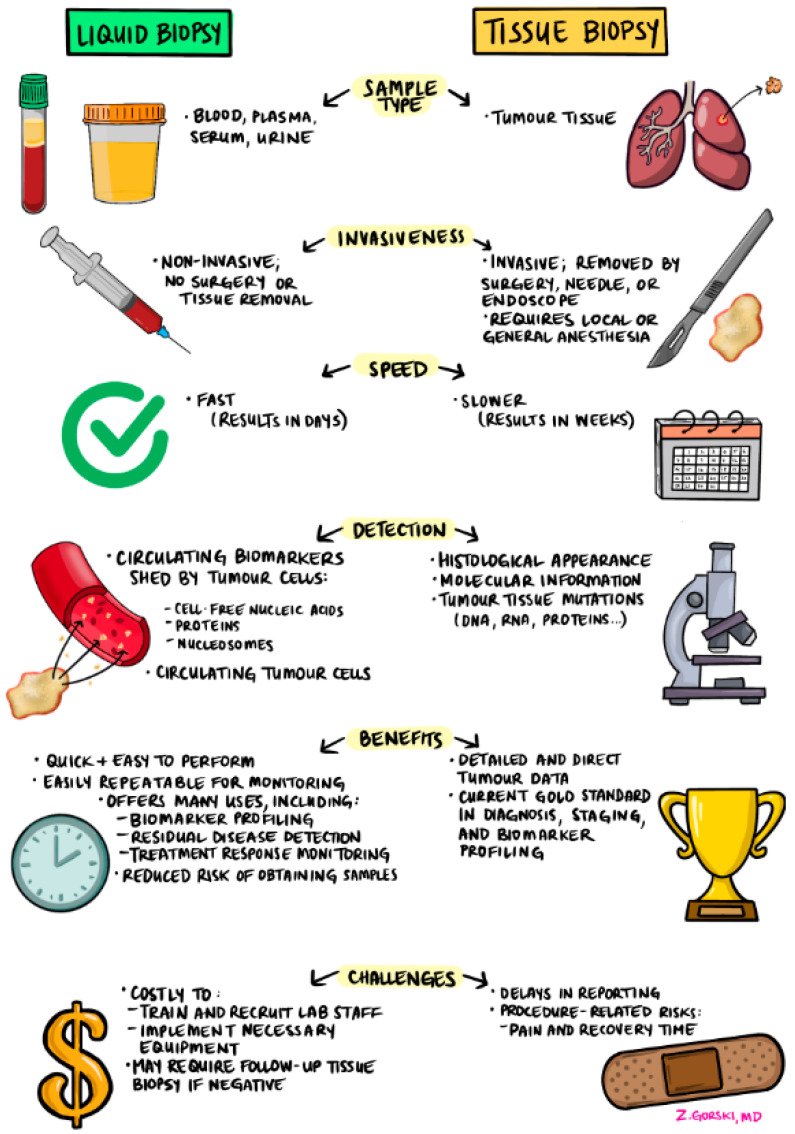
Comparison of liquid biopsy and tissue biopsy methods.

Other advantages of liquid biopsy over tissue testing include a reduced risk of obtaining samples in difficult-to-biopsy tumours such as pancreatic and prostate cancer. It also offers unique benefits in cases where tumours are insufficiently genotyped, presents lower costs, and has the potential to capture tumour heterogeneity from multiple anatomic sites [17,20]. In CRC (and potentially other cancer sites) liquid biopsy may be used in the adjuvant setting to identify minimal residual disease. Figure 2 summarizes the differences between liquid biopsy and tissue biopsy.

Liquid biopsy may also confer advantages when used in conjunction with tissue-based testing. A recent study of advanced NSCLC patients from Ontario, Canada showed that complementary use of liquid biopsy identified actionable alterations in more patients compared with tissue testing alone (68.5% versus 52.7%). The combination approach resulted in an incremental cost savings of $3,065 CAD per patient and a gain of 0.02 quality-adjusted life-years [21].

Against these benefits, the costs of implementing liquid biopsy, including support for training and recruitment of laboratory staff, acquisition of testing equipment, and ongoing operating costs present barriers to its implementation [22]. 

## 5. Toward Timely and Equitable Access to Biomarker Testing

Leading practices in improving access to biomarker testing were discussed at the conference as potential ways forward for Canada. These pertain to two main areas of concern for patients: (1) integration and coordination of services, and (2) health technology evaluation.

### 5.1. Integration and Coordination of Biomarker Testing Services

The onset of biomarker testing has had a transformational effect on laboratory medicine in Canada. Increasingly, this field is being viewed as an integral component in the clinical delivery of cancer care and as having significant impacts on health outcomes. The practice of laboratory medicine has expanded as some institutions now employ reflex testing as a standard of care. Pathology reports are now much more detailed than in the past, reflecting the complexity of testing and the need for interpretation and integration of information.

Laboratory medicine specialists attending the conference reported that their departments are now faced with challenges of recruiting and training qualified staff to meet these expanding roles. In this rapidly evolving field, national quality standards are called for with respect to testing procedures and in interpreting and communicating test results.

The following examples, from Canada and the UK, illustrate leading practices in improving efficiencies and quality of laboratory testing through integration on a regional and system-wide basis.

- Alberta’s laboratory system structure now includes all lab services, hospitals and public health systems on the same platform. Two provincial programs provide germline and somatic biomarker testing. Data on rare tests are consolidated, healthcare professionals access test results remotely, and reporting is standardized. This provincial model also allows for future automation.
- Quebec’s molecular genetics network, RQDM (Réseau québécois de diagnostic moléculaire), offers a comprehensive integrated approach by evaluating tests, organizing reimbursement, providing centralized implementation of testing, and having responsibility for provincial data collection. The system is of sufficient scale to allow investments in technology, the organization of testing and, as with Alberta, the potential to act as a portal for innovation.
- The United Kingdom’s National Genomic Test Directory, launched in April 2019, specifies which genomic tests are available for cancer and inherited rare diseases and corresponding patient eligibility criteria, identifies which medical specialties are able to order specific tests, and indicates who pays for the tests. In addition, a national genomic testing service is now delivered through a network of 7 regional Genomic Laboratory Hubs, each responsible for coordinating services for a particular part of the country [23].

Conference participants called for a national, cross-disciplinary professional body to develop a broadly-based framework for national standards regarding biomarker testing, reporting methods, and turnaround times. Obstacles to overcome include: heterogeneity across provinces with respect to funding and reporting strategies; mechanisms for adaptation in an environment of constant innovation; integration of hospital procurement systems; recruitment and training of laboratory specialists; standardization of test development and biomarker assay selection; data sharing; and a structured strategy for the evaluation of biomarkers. A cost-benefit analysis of CGP testing, currently in progress, will pave the way for these important changes.

### 5.2. Health Technology Assessment of Biomarker Tests

**Figure 3:**
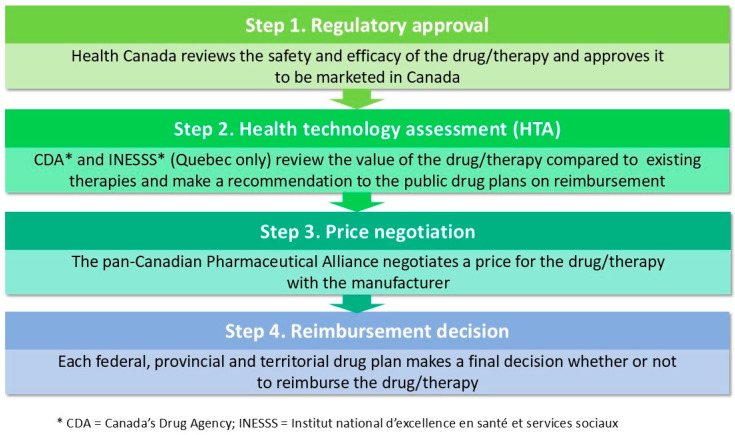
Drug reimbursement process in Canada. Diagram adapted from Ward et al. [24].

In Canada, reimbursement decisions for publicly-funded drugs and technologies follow a four-step process, as shown in Figure 3.

Canada’s Drug Agency (CDA, formerly the Canadian Agency for Drugs and Technologies in Health) conducts a health technology evaluation and makes recommendations to its member provinces (excluding Quebec). Each province then makes its own funding decision. Timely and equitable access to new biomarker tests is, therefore, largely dependent on utilizing an appropriate system of evaluation. 

Conference presenters called for the development of a system of evaluation and funding for biomarker testing that takes into account a broader scope of impacts on patients and across budgetary silos. For example, the cost of CGP may be offset by savings to drug budgets when precision medicines are prescribed and ineffective ones avoided. Likewise, better patient outcomes (especially in advanced and metastatic cancer) are predicted to lead to reductions in costs of hospital stays, which constitute one-third of public expenditures for cancer care [8]. Patient enrollment in clinical trials will likely increase as more patients’ genomes are profiled, allowing patients with advanced cancer greater access to experimental therapies (the costs of which are generally borne by manufacturers) and speeding up the drug development process. The implications of economic factors such as innovation and workforce participation should also be considered. 

Several approaches to evaluating and funding new health technologies were presented which promote equitable patient access to biomarker testing.

- The Australian government recently announced support for a AU$185 million innovative, multi-stakeholder, public-private partnership model for sustainable precision oncology, accelerating biomarker-dependent drug development by integrating clinical trials into the standard of care [25,26].
- A business case developed by the Collective Oncology Network for Exchange, Cancer Care Innovation, Treatment Access and Education (CONECTed), proposed a risk-sharing scheme inspired by successful access models developed in the HIV era. The process calls for early tentative approval based on Phase II trials and negotiated managed-entry agreements linked to availability of testing and collection of evidence of real world outcomes. Costs would be shared with manufacturers and adjusted over time according to whether the technology met expectations [27].
- Validated and transparent value frameworks, such as the one developed for Latin America by the Institute for Clinical Effectiveness and Health Policy (IECS) can provide guidance to address challenges [28].

## 6. Conclusions

The second Biomarkers Conference hosted by CCRAN, held in June 2024, built on the recommendations of the inaugural conference by exploring two main factors contributing to the lack of timely and equitable access to biomarker testing in Canada by patients with advanced/metastatic cancer: fragmentation of care and disparities in public funding among provinces and institutions. The emphasis was on access to CGP and liquid biopsy.

Conference participants identified challenges arising from the continued use of legacy processes and methodologies which are unsuited to the rapidly changing field of precision medicine. Examples from Canada and abroad illustrated successful, innovative approaches to health technology assessment (HTA) and integrated systems of coordination for biomarker testing.

Calls to action from conference participants included:

- Improve access to publicly-funded CGP for all patients with advanced/metastatic cancer by reforming HTA evaluation methodologies; and
- Develop and implement national standards for the performance, integration, coordination and communication of biomarker testing.

Achieving timely and equitable access to biomarker testing, and CGP in particular, will enable a precision medicine approach for the diagnosis, treatment and management of advanced/metastatic cancer which promises, in turn, to improve health outcomes and quality of life for the benefit of patients, their families, Canadians at large, the nation’s economy and health system. 

## Figures and Tables

**Table 1:** Results of patient survey on access to biomarker and CGP testing in Canada.

Challenges and Missed Opportunities	Patient Responses
Lack of awareness of the importance of testing	The majority of patients reported that their oncologists had not initiated discussions about biomarker testing (biomarkers 75%, CGP 77%)
Delays in obtaining test results	42% and 43% of tested patients waited over 4 weeks for the results of their biomarker and CGP tests, respectively
Positive impacts on treatment planning	Biomarker testing results changed treatment plans for 16% of those who had already begun treatment. Of these, 93% included a precision medicine based on the patient’s tumour’s biomarker status
Benefits of post-biomarker testing on patient outcomes	Increased overall survival (67% of respondents) Increased progression-free survival (33%) Elimination of cancer (13%) Improvements in symptoms and/or reduced side effects (60%)
Systemic barriers to access to CGP testing	65% of patients who had CGP testing experienced challenges due to: Lack of provincial CGP coverage (30%) CGP unavailable through their cancer centre (27%) Overall coordination of health services (24%) Financial barriers (16%)

## Data Availability

The data presented in this report are available within this article.

## Appendix A

### Appendix A.1. Conference Organization

The 2024 Biomarkers Conference was organized under the auspices of the Colorectal Cancer Resource & Action Network (CCRAN), which provides colorectal cancer patient and caregiver support, education and advocacy in Canada, with an expanded mandate to work collaboratively with multiple tumour type patient advocacy groups to reduce the burden of cancer in Canada.

An Expert Steering Committee provided direction on the conference objectives, agenda, speakers and invited participants. Committee members were:

- Chair: Sharlene Gill, Medical Oncologist, BC Cancer Agency, Vancouver, BC
- Brandon Sheffield, Pathologist, William Osler Health System, Brampton, ON
- Ravi Ramjeesingh, Medical Oncologist and Lead for Hepato-pancreato-biliary (HPB) Cancer, Nova Scotia Cancer Centre, Halifax, NS
- Christine Brezden-Masley, Medical Oncologist, Breast & Gastrointestinal, Mount Sinai Hospital, Toronto, ON
- Fred Horne, Health Policy Advisor, Edmonton, AB
- Neesha Dahni, Medical Oncologist, Gynecologic Cancer, Princess Margaret Cancer Centre, Toronto, ON
- Shaqil Kassam, Medical Oncologist (Lung), Southlake Stronach Regional Cancer Centre, Newmarket, ON
- Allen Chankowsky, Patient expert, Toronto, ON
- Robin McGee, Patient expert, Port Williams, NS

### Appendix A.2. Participation

Participants included 52 expert speakers from Canada, the United States, Europe and Australia; patient advocacy group leaders representing multiple tumour sites; and 553 registrants from 22 countries.

### Appendix A.3. Agenda

The meeting agenda is presented in Table A1. All sessions were held virtually.

**Table A1:** Conference agenda.

Session	Speakers
Day 1: Thursday, 20 June 2024	
Conference Opening–Day 1	Dr. Monika Slovinec D’Angelo, Chief Research Officer, CCRAN
Welcome from CCRAN’s President	Ms. Filomena Servidio-Italiano, President & CEO, CCRAN Mr. Allen Chankowsky, Patient expert
Barriers and Unequal Access to Timely Molecular Testing Results: Addressing the Inequities in Cancer Care Delays Across Canada	Dr. Stephanie Snow, President, Lung Cancer Canada
Results of a National Pan-Tumour Survey Regarding Access to Biomarker Testing Results: What are Canadian Advanced Cancer Patients Saying?	Dr. Monika Slovinec D’Angelo, Chief Research Officer, CCRAN Ms. Cassandra Macaulay, Senior Manager of Programs & Education, CCRAN Ms. Filomena Servidio-Italiano, President & CEO, CCRAN
Collaborative Advocacy Strategies for Addressing Biomarker Testing Access Issues Across Tumour Types	*Patient Group Roundtable* Moderator: Ms. Martha Raymond, Founding Executive Director, GI Cancers Alliance Inc. (New York, NY, USA) Patient Organizations: Ms. Teresa Norris, Founder and President, HPV Global Action Ms. Richelle Laturnus, Support Services Manager, Prostate Cancer Foundation Canada Mr. Ken Noel, Executive Director, The Walnut Foundation Mr. Allen Chankowsky, Patient Advocate Ms. Martine Elias, Executive Director, Myeloma Canada Ms. Brenda Clayton, Founder, Cholangio-Hepatocellular Carcinoma Canada Ms. Maura Cosgrave, Childhood Cancer Canada
Tackling the Implementation Gap for the Uptake of Advanced Molecular Testing into Healthcare Systems: What are the Priorities?	*Panel Session* Moderator: Dr. Yvonne Bombard, Canada Research Chair in Genomics Health Services and Policy Expert Panelists: Dr. Denis Horgan, Executive Director, European Alliance of Personalised Medicine (EAPM) Dr. Laura Weeks, Director, Health Technology Assessment, Canada’s Drug Agency (formerly Canadian Agency for Drugs and Technologies in Health) Dr. Alan Spatz, Chief, Department of Clinical laboratory medicine, McGill University Health Center (MUHC) Dr. Michael Mengel, Chair & Medical Director, Laboratory Medicine & Pathology, University of Alberta Mr. Craig Ivany, Chief Provincial Diagnostic Officer, Provincial Health Services Authority (PHSA), British Columbia Dr. Harriet Feilotter, Division Head, Genome Diagnostics, Laboratory Medicine Program, University Health Network, Toronto, Ontario
Delivering on the Promise of Precision Oncology: Efficacy and Turn-Around Time for Biomarker Testing	*Presentations & Panel Session* Moderator: Dr. Shaan Dudani, Medical Oncologist, William Osler Health System, Ontario Expert Panelists: Ms. Emmanuelle Langelier, Caregiver to rectal cancer patient Dr. Brandon Sheffield, Medical Director of Advanced Diagnostics, William Osler Health System, Ontario Dr. Harriet Feilotter, Division Head, Genome Diagnostics, Laboratory Medicine Program, University Health Network Dr. Stephen Yip, Clinician-Scientist, Neuropathologist, BC Cancer, Vancouver Dr. Michael Carter, Medical Director, Molecular Diagnostics, Nova Scotia Health Dr. Nicola Normanno, Scientific Director, Istituto Romagnolo per lo Studio dei Tumori (IRST) “Dino Amadori”, Istituto di Ricovero e Cura a Carattere Scientifico (IRCCS), Meldola, Italy
The Utility and Feasibility of Comprehensive Genomic Profiling (CGP) in Clinical Practice	*Clinician Roundtable* Moderator: Dr. Christopher Lieu, Director, GI Oncology, University of Colorado Expert Panelists: Dr. Michael Raphael, Medical Oncologist, Odette Cancer Centre, Sunnybrook Health Sciences Centre, Toronto Dr. Christine Brezden-Masley, Director, Marvelle Koffler Breast Centre, Sinai Health System, Toronto Dr. Shaqil Kassam, Medical Oncologist, Southlake Stronach Regional Cancer Centre, Newmarket, Ontario Dr. Neesha Dhani, Medical Oncologist, Gynecologic Cancers Disease Site Group, University Health Network-Princess Margaret Cancer Centre, Toronto Dr. Ravi Ramjeesingh, Chair, Hepato-Pancreato-Biliary (HPB) Cancer Disease Site Group, Nova Scotia Cancer Centre, Halifax
Day 1 Closing Remarks	Ms. Filomena Servidio-Italiano, President & CEO, CCRAN
DAY 2: Friday, 21 June 2024	
Conference Opening	Dr. Monika Slovinec D’Angelo, Chief Research Officer, CCRAN
Welcome from CCRAN’s President-Day 2	Ms. Filomena Servidio-Italiano, President & CEO, CCRAN Ms. Laura Greer, Patient Expert
Equitable Access to Genomic Medicine in Canada: Bridging the Divide Between What Could be and What is through Investment and Communication Strategies	*Presentations:* Ms. Laura Greer, National Health Sector Lead, Health and Wellness, Hill & Knowlton Canada Dr. Catalina Lopez-Correa, Chief Scientific Officer, Genome Canada
The Utility and Feasibility of ctDNA Testing for Minimal Residual Disease (MRD) in Optimizing Cancer Care in Adjuvant & Metastatic Disease Settings	*Clinician Roundtable:* Moderator: Dr. Sharlene Gill, Medical Oncologist, BC Cancer, Vancouver *Expert Panelists:* Dr. Pashtoon M. Kasi, Oncologist and Precision Medicine Director for Liquid Biopsy Research, Weill Cornell Medicine, Englander Institute of Precision Medicine, New York, USA Prof. Klaus Pantel, Chairman, Institute of Tumor Biology, University Medical Center Hamburg-Eppendorf, Germany Dr. Christine Brezden-Masley, Medical Director, Cancer Program, Sinai Health System, Toronto Dr. Michael Raphael, Medical Oncologist, Odette Cancer Centre, Sunnybrook Health Sciences Centre, Toronto Dr. Joao Paulo Solar Vasconcelos, Gastrointestinal Medical Oncology Fellow, BC Cancer, Vancouver Dr. Ceilidh MacPhail, Medical Oncologist, Nova Scotia Cancer Centre
Research into Practice: Biomarker Driven Patient Education Tools	*Presentations:* Dr. Yvonne Bombard, Canada Research Chair in Genomics Health Services and Policy Ms. Filomena Servidio-Italiano, President & CEO, CCRAN Dr. Mary De Vera, Tier 2 Canada Research Chair in Health Outcomes and Treatment Adherence
Examining Policy Recommendations for Accelerating Access to Precision Medicine through a Value-Based Healthcare Lens	Panel Session: Moderator: Dr. Monika Slovinec D’Angelo, Chief Research Officer, CCRAN Expert Panelists: Mr. Fred Horne, Principal, Horne and Associates Health Policy Consultants and former Alberta Minister of Health Dr. Darren Larsen, Chair, Cancer Quality Council of Ontario Dr. David J. Stewart, Professor of Medicine, Division of Medical Oncology, University of Ottawa and The Ottawa Hospital Ms. Jenn Gordon, Lead, Strategic Operations and Engagement, Rethink Breast Cancer
The Interplay Between Genomic Profiling and Real-World Evidence in Precision Medicine: Optimizing Cancer Care and Facilitating Payer Decisions	*Panel Session:* Moderator: Dr. Monika Slovinec D’Angelo, Chief Research Officer, CCRAN *Expert Panelists:* Ms. Louise Binder, Health Advocate and Health Policy Consultant, CONECTed-Save Your Skin Foundation Dr. Shaqil Kassam, Medical Oncologist, Southlake Stronach Regional Cancer Centre, Newmarket, Ontario Dr. Jason Karamchandani, President, Canadian Association of Pathologists Ms. Sang Mi Lee, Executive Director, MORSE Consulting Inc.
Feasible Opportunities to Adopt Comprehensive Genomic Profiling in Canada: An International Perspective	*Panel Session:* Moderator: Mr. Don Husereau, Adjunct Professor of Medicine, University of Ottawa *Expert Panelists:* Prof. David M. Thomas, Director, Centre for Molecular Oncology, University of New South Wales, Sydney, Australia Dr. Shantanu Banerji, Director, Precision Oncology and Advanced Therapeutics, CancerCare Manitoba Dr. Cathy Eng, David H. Johnson Chair in Surgical and Medical Oncology, Vanderbilt-Ingram Cancer Center, Nashville, Tennessee Prof. Dr. Patrick Pauwels, Head of the Laboratory of Molecular Pathology & Co-Director of the Center of Oncology Research (CORE), Antwerp University, Belgium
Closing Remarks	Ms. Filomena Servidio-Italiano, President & CEO, CCRAN
